# Coexisting Medullary and Papillary Thyroid Carcinomas: A Case of Dual Neoplasia With a High Risk of Misdiagnosis

**DOI:** 10.7759/cureus.71581

**Published:** 2024-10-16

**Authors:** Santiago Sierra Castillo, Maria A Henao Rincón, David Aristizabal Colorado, David Alexander Vernaza Trujillo, Alin Abreu Lomba

**Affiliations:** 1 Medicine, CES University, Medellín, COL; 2 Otolaryngology, Universidad de Cartagena, Cartagena, COL; 3 Internal Medicine, Interinstitutional Group on Internal Medicine 1 (GIMI1), Universidad Libre, Cali, COL; 4 Epidemiology, Fundación Universitaria del Área Andina, Bogotá, COL; 5 Epidemiology and Public Health, Interinstitutional Group of Internal Medicine 1 (GIMI1), Universidad Libre, Cali, COL; 6 Endocrinology, Imbanaco Clinic, Cali, COL

**Keywords:** cervical tumor, medullary cancer, papillary cancer, surgery, thyroid carcinoma presentation

## Abstract

The simultaneous occurrence of more than two types of neoplasms is rare due to their significant phenotypic differences. Thyroid carcinoma is regularly associated with genetic alterations and endocrine syndromes. However, the etiology of the forms of papillary thyroid carcinoma (PTC)/medullary thyroid carcinoma (MTC) is still not fully understood.

We present the case of a 60-year-old male with no significant history of thyroid disease who presented with dysphonia. Left vocal cord paralysis and a cervical tumor lesion were observed. A mixed medullary/papillary carcinoma was diagnosed. Surgical resection of the mediastinal tumor and hemithyroidectomy revealed a poorly differentiated blue cell MTC, with a tumor size of 6.5 x 6.4 x 4.4 cm and a Ki-67 proliferation index of 10%. The tumor was positive for cytokeratin AE1/AE3, carcinoembryonic antigen, synaptophysin, chromogranin, thyroid transcription factor 1 (TTF-1), S-100, and calcitonin. Metastasis was identified in a lymph node at the mediastinum, supporting a diagnosis of usual-type papillary thyroid carcinoma, with positivity for cytokeratin AE1/AE3, thyroglobulin, and TTF-1. Genetic tests related to hereditary cancer (Gencell Pharma, Bogotá, Columbia) were negative.

The simultaneous presence of MTC and PTC in a patient is a rare event. The clinical characteristics and biological behavior of these cancer types can vary. The prognosis is directly related to the stage at presentation and the condition at the time of diagnosis.

## Introduction

The prevalence of papillary thyroid carcinoma (PTC) accounts for 85% of all thyroid cancer cases [1]. In contrast, medullary thyroid cancer ranks second in terms of frequency, constituting approximately 10% of thyroid neoplasms [2]. The simultaneous occurrence of both neoplasms is uncommon, not only due to their phenotypic differences but also because they arise from distinct pathophysiological mechanisms, with no common molecular pathway. Since 1980, the mortality rate has remained constant, averaging between 0.4 and 0.5 per 100,000 people annually [1]. Medullary thyroid carcinoma (MTC) originates from C cells or parafollicular cells and currently accounts for 4% to 8% of all thyroid cancers [3].

Thyroid carcinoma is frequently associated with genetic alterations and endocrine syndromes, often being a component of multiple endocrine neoplasia type 2 (MEN 2), an autosomal dominant hereditary condition, or familial MTC. It has been demonstrated that point mutations activating the REarranged in Transfection (RET) proto-oncogene are associated with this phenomenon [1, 4]. Regarding patient outcomes, those diagnosed with PTC exhibit a higher 10-year relative survival rate, reaching 0.98. Conversely, the prognosis for MTC is generally less favorable compared to that of PTC, with a survival rate of 0.80 [2].

Two patterns of presentation may manifest which are as follows: mixed medullary follicular thyroid carcinoma (MMFTC), where both components coexist in the same neoplastic nodule, and PTC/MTC, where the two components are separated by healthy thyroid tissue [5]. In a review conducted between 2001 and 2017, only five case reports of PTC/MTC were identified [6]. The objective of this report is to describe a case of this rare association.

## Case presentation

We present the case of a 60-year-old male patient with no family history of thyroid disease, although he had a previous history of benign prostatic hyperplasia, which led to an open prostatectomy in 2012. There were no records of head or neck irradiation, nor were there any personal or family histories related to endocrine neoplasms or other pathologies. The patient presented with a three-week history of dysphonia, not associated with pain, fever, or additional systemic symptoms.

**Figure 1:**
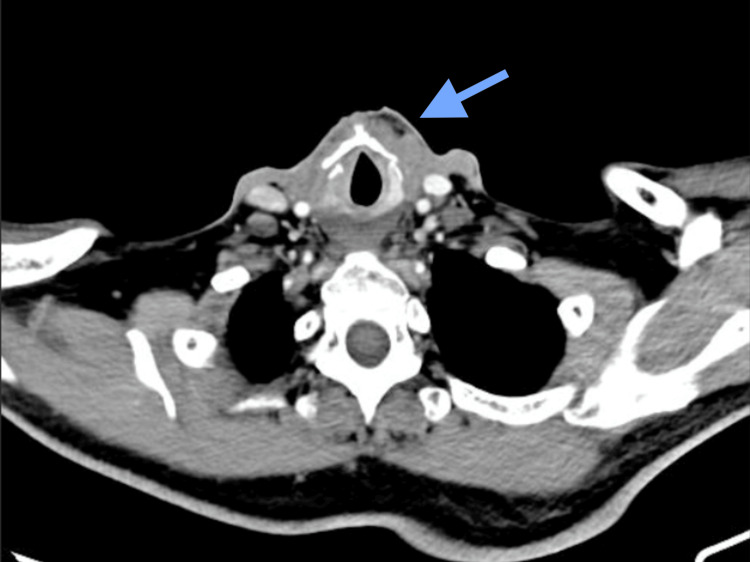
Right lateral deviation of the trachea secondary to a 72 x 63 x 56 mm expansile, tumor-like lesion, with no signs of dependence on the thyroid gland, presenting irregular, infiltrative borders and showing heterogeneous enhancement following contrast administration

On physical examination, the patient's vital signs were within normal limits, with no evident motor or sensory deficits. No significant findings were detected during the head and neck examination. A flexible nasolaryngoscopy, along with laryngeal stroboscopy, was performed, revealing left median vocal cord paralysis. Computed tomography (CT) of the neck revealed a right lateral deviation of the trachea, resulting from an expansive tumor-like lesion at the left paratracheal level. This lesion, measuring 72 x 63 x 56 mm, presented irregular and infiltrative borders with heterogeneous enhancement following contrast administration (Figure 1). It is noteworthy that there were no signs suggesting a connection to the thyroid gland, although the thyroid appeared homogeneous, with the left lobe displaced apically.

**Figure 2:**
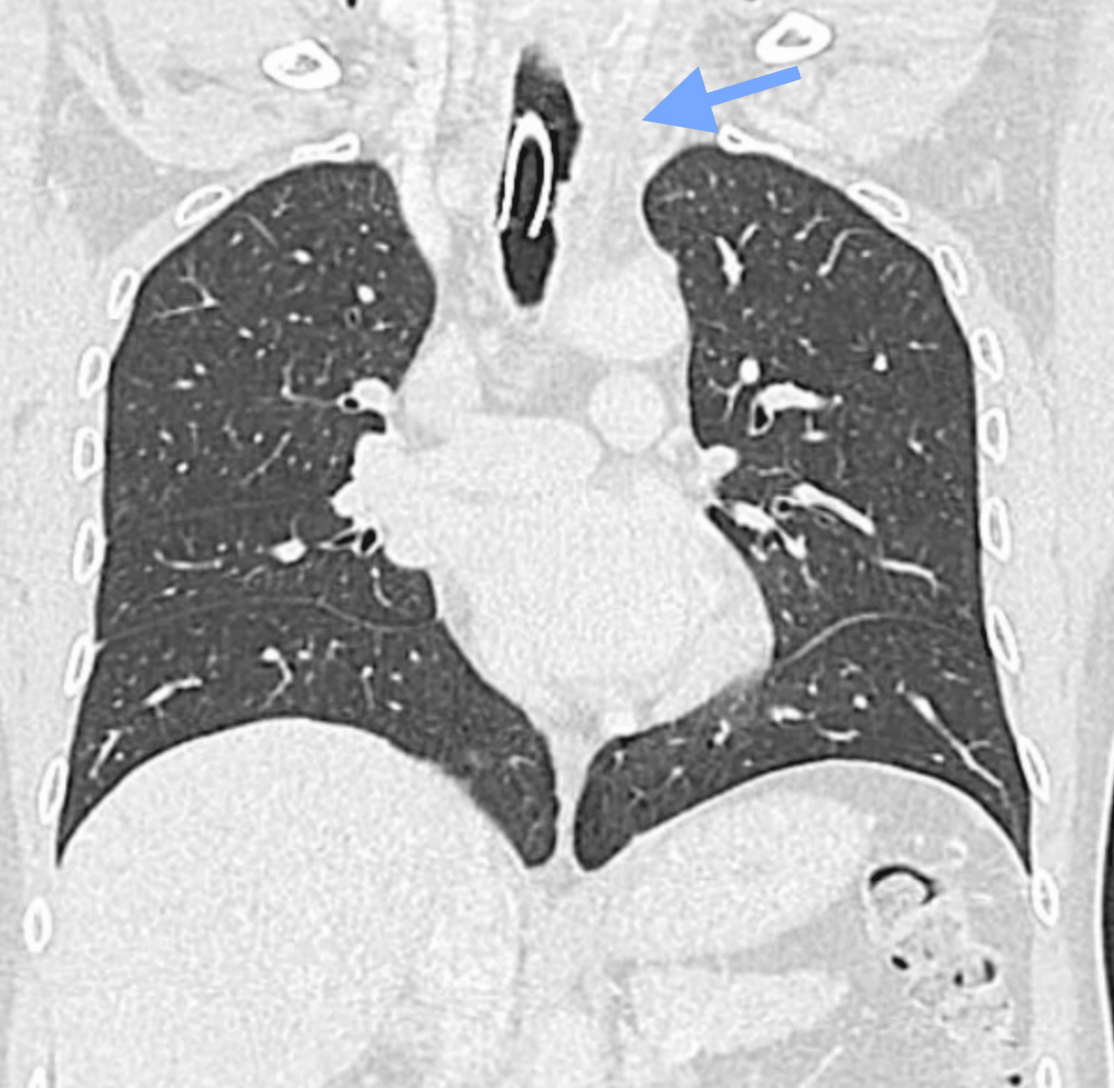
At the left paratracheal level in the middle mediastinal region, there is a 60 x 72 x 54 mm expansile, tumor-like lesion with signs of angiogenesis and polylobulated contours, extending towards the thoracic inlet (arrow).

Subsequently, the patient underwent an abdominal CT scan, which revealed focal thickening of the cecum with a nodular morphology measuring 16 x 14 mm. Additionally, post-surgical changes were observed in the prostate, with no evidence of inguinal, internal, or external iliac adenopathy suggestive of a tumorous process, nor at the retroperitoneal level. Thoracic CT revealed a large tumor-like expansive lesion in the left paratracheal region of the middle mediastinum, showing signs of angiogenesis and polylobulated contours (Figure 2). This lesion, measuring 60 x 72 x 54 mm, displaced the trachea contralaterally, extending towards the thoracic inlet.

A colonoscopy was performed, which revealed the presence of generalized diverticulosis and grade II internal hemorrhoids. The esophageal study via esophagogastroduodenoscopy (EGD) showed extrinsic compression of the cervical esophagus without mucosal involvement. Given the diagnostic suspicion, a mediastinoscopy was performed, which reported mediastinal tumor infiltration by a small blue cell malignant neoplasm. To confirm the diagnosis, positron emission tomography-computed tomography (PET-CT) was requested by the head and neck department, which reported pathological hypermetabolism in the cervical mass extending to the superior mediastinum with no evidence of thyroid gland involvement. The lesion, measuring 61 x 69 x 77 mm, was consistent with a primary neoplastic lesion. No other hypermetabolic lesions suggestive of a neoplastic process were identified in other areas of the body.

The decision was made to proceed with surgery for the resection of the left mediastinal tumor, followed by a left hemithyroidectomy. Subsequently, a second revision surgery was performed, during which a right hemithyroidectomy, lymph node dissection, and tracheostomy were carried out due to bilateral recurrent laryngeal nerve involvement and the high risk of ventilatory insufficiency. The tumor, with stony characteristics and measuring 8 x 4 cm, infiltrated cervical muscle fibers and the left lobe of the thyroid. The pathological report revealed a poorly differentiated malignant tumor, with MTC as the primary possibility. The tumor size was 6.5 x 6.4 x 4.4 cm, located less than 1 mm from both the peripheral and perimetral margins. It was classified as MTC, cT4a (involvement of trachea, esophagus, and left recurrent laryngeal nerve), N1b (metastasis in left cervical levels), and M0, clinical stage IVA. The resection was classified as R2, with residual tumor towards the esophagus.

Immunohistochemical analysis of the mediastinal mass and the right thyroid lobe identified a mediastinal tumor consistent with a poorly differentiated small blue cell malignant neoplasm. This tumor showed positivity for cytokeratin AE1/AE3, carcinoembryonic antigen, synaptophysin, chromogranin, TTF-1, S-100, and calcitonin, indicating medullary thyroid carcinoma with a Ki-67 proliferation index of 10%. Metastasis was identified in a lymph node, supporting the diagnosis of usual-type metastatic papillary thyroid carcinoma, with positivity for cytokeratin AE1/AE3, thyroglobulin, and thyroid transcription factor 1 (TTF-1), confirming usual-type metastatic papillary carcinoma. In conclusion, a diagnosis of concomitant mixed medullary/papillary carcinoma was established, revealing both histological types in the same patient. Next-generation sequencing (NGS) for genes related to hereditary cancer (Gencell Pharma, Bogotá, Columbia) was negative.

Postoperative control examinations, specifically PET-CT, did not reveal clear evidence of viable tumors detectable by this technology. Post-surgical changes were observed in the middle third of the sternum, with the presence of a soft tissue mass in the posterior region, which should be evaluated in the clinical context, considering the patient's history. The laboratory results are as follows: E-thyroid stimulating hormone (TSH): 0.8 uIU/mL, free thyroxine (T4): 1.4 ng/mL, anti-thyroglobulin antibodies: 150 IU/mL, thyroglobulin <0.14 ng/mL, parathyroid hormone (PTH): 27 pg/mL (normal range: 15-65), calcitonin: 159 pg/mL (normal range: <11.8), calcium: 1.16 mg/dL (normal range: 1.15-1.33), phosphorus: 4.0 mg/dL.

After evaluation by the head and neck and oncology teams, it was determined that the patient had undifferentiated thyroid carcinoma, specifically MTC. The cancer stage was classified as pT4a, with a tumor size of 6.5 cm and involvement of the trachea, esophagus, left recurrent laryngeal nerve, and left recurrent laryngeal nerve. Regarding the lymph nodes, pN1a was recorded, with four out of 10 nodes affected in level 6 of the left neck. No distant metastases were observed (M0, negative chest CT), and the stage was considered IVA. It was suggested that the surgical resection was incomplete (R2) with residual tumor towards the esophagus. The patient was considered a candidate for intensity-modulated radiotherapy (IMRT), with a total of 30 fractions on the thyroid tumor bed. Currently, the patient is tracheostomy- and gastrostomy-dependent, with a Barthel index of 100/100. The latest PET-CT reports a new lesion consistent with a solitary nodule of recent onset with moderate-intensity metabolic changes within it, located in the lingular region of the left lower lung lobe. The patient is considered a candidate for treatment with lenvatinib.

## Discussion

The manifestation of thyroid cancer of both PTC and MTC types together presents a notable rarity in its presentation. This also raises the possibility of a debate about whether their occurrence is due to a fortuitous coincidence or a specific genetic alteration that facilitates their development [5,7]. The prevalence of the simultaneous occurrence of both types of thyroid cancer is 0.28% of all cases [5,8]. The coexistence of PTC and MTC may manifest independently, either as a collision or mixed tumor, presenting dual differentiation. The latter is classified as mixed medullary and papillary carcinoma according to the World Health Organization (WHO) classification, which is extremely rare [5]. In our case, lesions with MTC and PTC attributes were observed in two different locations.

Some case series reviews indicate that the age of presentation for thyroid cancer ranges between 27 and 70 years. There is a slight prevalence in male patients compared to females, with presentation reports in a ratio of 1.2:1 and 2:1, respectively [9, 10]. The PTC/MTC combination does not alter the epidemiological, pathological, or specific clinical manifestations of the different forms of cancer; the prognosis is primarily linked to the stage at which MTC is detected at the time of diagnosis [6].

Hypoechoic nodules with poorly defined or well-defined sonographic margins have been evidenced [9]. Fine-needle aspiration cytology (FNAC) generally confirms the diagnosis of PTC or suspicious carcinoma. In situations where a combination of MTC and PTC is present, it is argued that FNAC cytology has limited diagnostic value, as it is challenging to detect both pathological types of thyroid cancer [10].

Since the specific histological features of PTC, such as nuclear clearing, nuclear grooves, and pseudoinclusion with papillary architecture, are well known, cytopathologists often encounter this entity due to its high prevalence. In contrast, MTC can exhibit a wide range of histological morphology in terms of both architecture and cytological characteristics. Certain cytological features, such as eccentric nuclei, salt-and-pepper chromatin, inconspicuous nucleoli, binucleation or multinucleation, and poorly defined cytoplasmic borders, have been considered pathognomonic findings in the case of MTC [3, 5]. In this patient, it was determined that the first mediastinal tumor was constituted by a poorly differentiated small blue cell malignant tumor, arranged in an organoid pattern with salt-and-pepper chromatin in nests and cords.

The identification of elevated serum calcitonin levels above normal values facilitates the diagnosis of MTC [11]. In patients with normal calcitonin levels, the diagnosis can be established by measuring calcitonin in the fluid obtained during the aspiration of a suspicious thyroid nodule [12]. Additionally, serum carcinoembryonic antigen (CEA) emerges as an additional reliable indicator for MTC, with elevated CEA levels indicating the possibility of distant metastases, as was the case in the reported patient [4]. Positivity for cytokeratin, synaptophysin, chromogranin, TTF-1, S-100, and calcitonin focused the diagnosis on MTC, similar to other studies that have used immunohistochemistry to assess levels of calcitonin, CEA, synaptophysin, and chromogranin A, revealing positive results for TTF-1 and paired-box gene 8 (PAX8), while thyroglobulin (TG) was negative [13]. Regarding the PTC type, the immunohistochemistry of these types of samples was positive for TTF-1, PAX8, and TG and negative for calcitonin, synaptophysin, and chromogranin [13].

In terms of treatment, it is essential to address both MTC and PTC [10]. Total or near-total thyroidectomy is considered the standard therapeutic modality for both types of cancer [9, 14]. Due to the unfavorable prognosis associated with MTC, it becomes crucial to perform surgical intervention to control this condition [5, 6, 15]. The extent of lymph node dissection is determined based on preoperative calcitonin levels. The treatment strategy was similar to those recommended by various guidelines. In our case, resection of the mediastinal tumor was proposed, followed by a hemithyroidectomy, lymph node dissection, and tracheostomy. Calcitonin levels exceeding 20, 50, 200, and 500 pg/ml are associated with the presence of metastases in different areas of the lymph nodes, such as the central and lateral ipsilateral neck, the contralateral central neck, the contralateral lateral neck, and the superior mediastinum, respectively [3, 4, 6, 15].

Given that patients presenting with mixed thyroid cell tumors along with papillary thyroid tumors exhibit characteristics of both tumor types, it is feasible to approach their postoperative treatment through the application of radioactive iodine therapy, endocrine therapy, and specific pharmacological therapy as proposed after the intervention in this case, considering that the tumor resection was not complete, and due to the damage to several structures, IMRT-type radiotherapy was chosen. Some studies suggest the possibility of treating patients with advanced disease through a combination of radiotherapy and chemotherapy, aiming to achieve local remission of the lesion; however, extensive data sets are still needed to conclusively support this hypothesis [4]. Some reviews have demonstrated that the survival of patients with mixed carcinoma may be better than that of those with exclusively medullary-type carcinomas [10, 16].

Patients with mixed thyroid cell tumors should be evaluated following the specific monitoring principles for each tumor type, as there is no clear standardization for managing mixed tumors. The focus is on the detection of serum calcitonin, carcinoembryonic antigen, and serum TG, along with a review of thyroid function and cervical ultrasound. When necessary, additional procedures such as CT and PET are suggested to evaluate recurrences or metastases [4].

## Conclusions

The coexistence of papillary and medullary carcinoma within the same thyroid gland is uncommon but notable, with an increase in its incidence observed over the past three decades. Early diagnosis of MTC continues to be a critical challenge, whether it presents in isolation or association with another type of thyroid cancer, such as papillary carcinoma. The prognosis is directly related to the stage at presentation and the condition at the time of diagnosis. Larger, prospective studies with more detailed molecular investigations are needed to better understand this still uncommon presentation.
